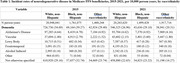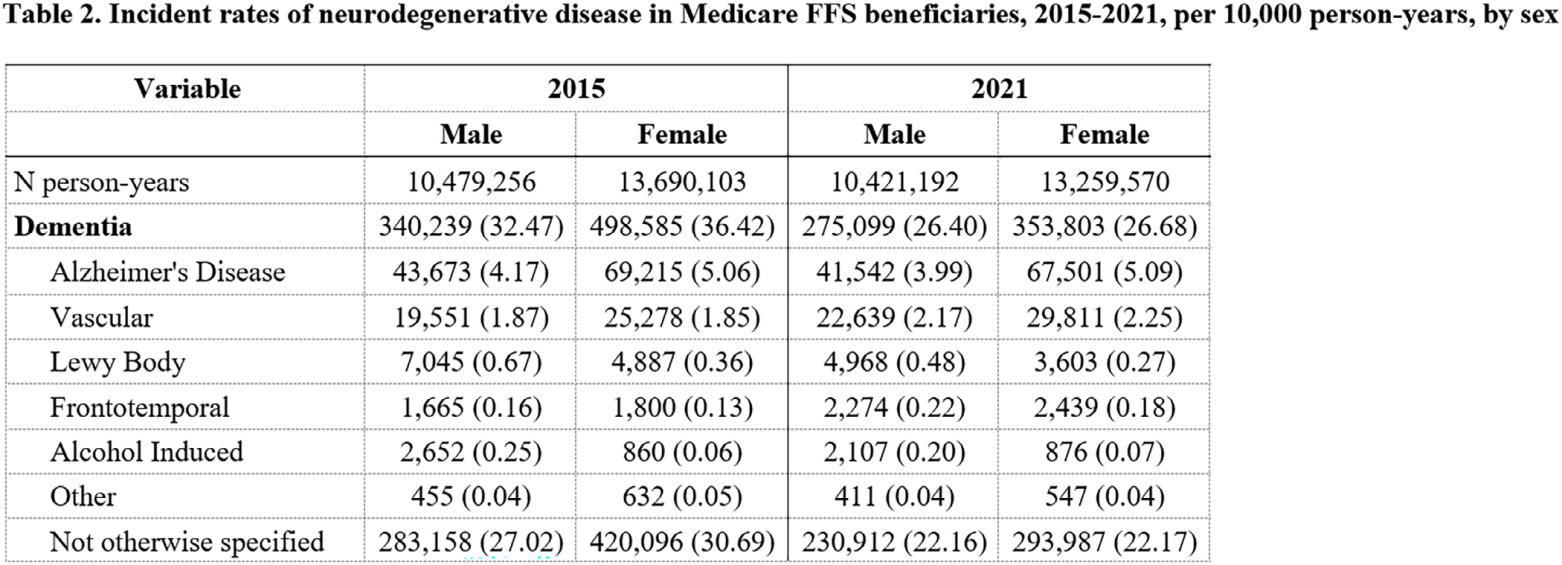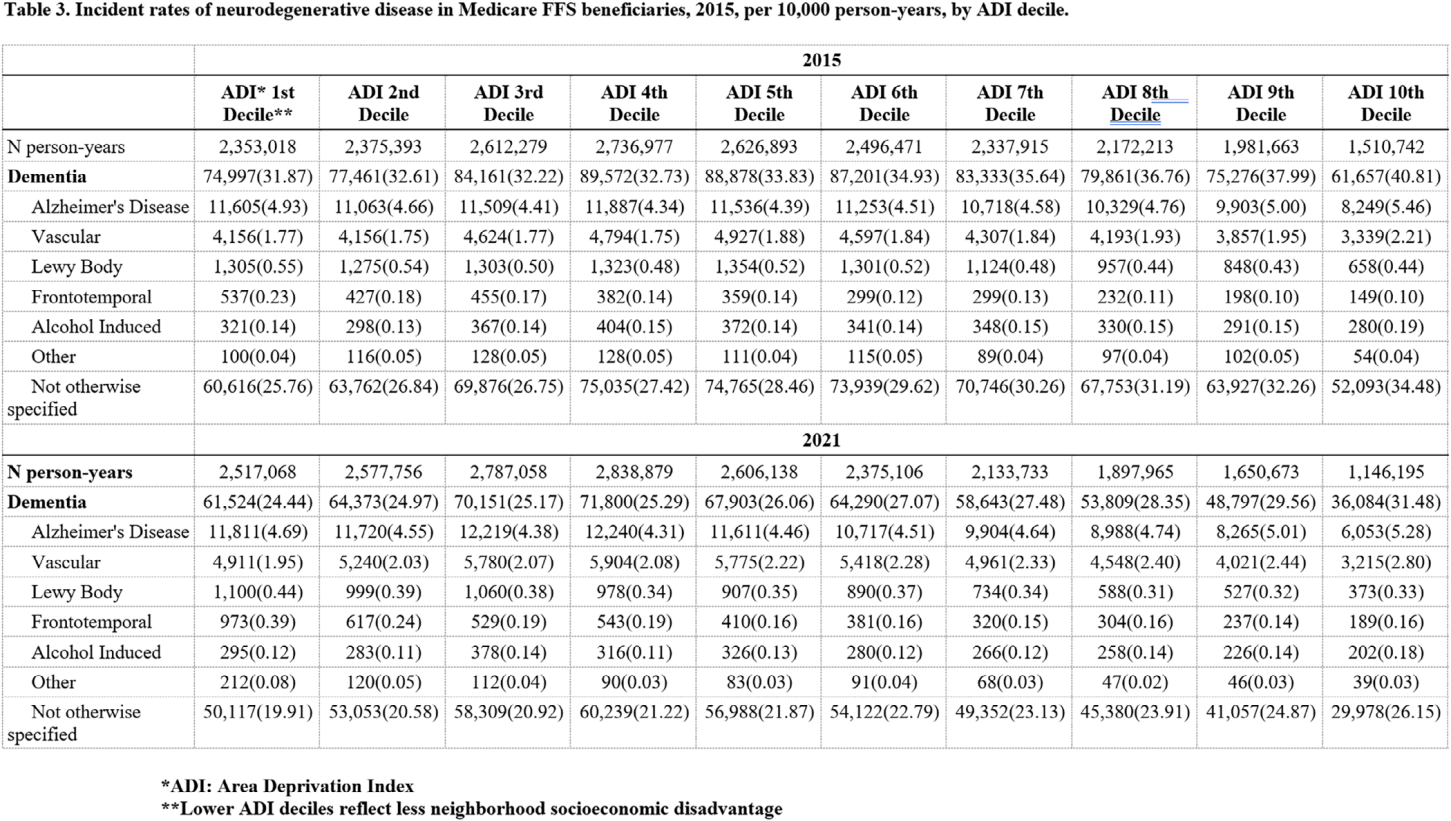# Trends in Diagnosis of Dementia Subtypes in Nationwide Medicare Claims, 2015‐2021, by Race/Ethnicity, Sex, and Neighborhood Socioeconomic Status

**DOI:** 10.1002/alz.093412

**Published:** 2025-01-09

**Authors:** Jay B Lusk, Cassie B Ford, Beau Blass, Kim G Johnson, Amy G Clark, Samir Soneji, Richard J O'Brien, Bradley G Hammill, Emily C O'Brien

**Affiliations:** ^1^ Duke University, Durham, NC USA

## Abstract

**Background:**

It is not well understood how incidence patterns of subtypes of Alzheimer’s disease and related dementias (ADRD) have evolved in real‐world practice. While cohort and brain bank studies provide precise biological definition of ADRD subtypes, these populations may not be representative and may not reflect how dementia is coded and diagnosed in routine clinical practice. Therefore, we sought to perform a nationally representative study of medical claims data to understand trends in diagnosis of dementia by dementia subtypes in routine clinical practice.

**Method:**

We studied 100% of nationwide Medicare claims from 2014‐2021 and evaluated dementia diagnoses by specified subtypes with associated international classification of diseases (ICD) codes (Alzheimer’s disease, vascular, Lewy body, frontotemporal, alcohol induced, and not otherwise specified). We stratified results by age, sex, race/ethnicity, and neighborhood socioeconomic deprivation measured by the area deprivation index, a well‐established score that describes neighborhood socioeconomic conditions. Incidences are presented as number of new cases per 10,000 person‐years. Race/ethnicity were grouped as White, non‐Hispanic; Black, non‐Hispanic; and other race/ethnicity, concordant with other Medicare claims data analyses.

**Result:**

A total of 5,721,711 patients with incident dementia were included in the study. Dementia diagnoses were more frequently coded as vascular in 2021 than 2015 (1.85 cases per 10,000 person‐years in 2015 vs 2.21 cases per 10,000 person‐years in 2021) and less frequently coded as not otherwise specified (29.1 cases per 10,000 person‐years in 2015 vs 22.17 cases per 10,000 person‐years in 2021). Coding by dementia subtype varied by race (Table 1) and sex (Table 2). Dementia diagnoses were much more frequently coded as “not otherwise specified” among beneficiaries from socioeconomically deprived neighborhoods (34.48 per 10,000 person‐years in the most deprived decile vs 25.76 in the least‐deprived decile) (Table 3).

**Conclusion:**

In a national database of Medicare claims, there were substantial secular trends and differences in coding of dementia subtypes by race/ethnicity, sex, and neighborhood socioeconomic status. Secular trends in coding could reflect variation in health system performance of accurate diagnosis of dementia subtypes. Future studies should correlate claims data classifications with confirmed neuropathological classifications.